# How to Test Mandatory Text Templates: The European Patient Information Leaflet

**DOI:** 10.1371/journal.pone.0139250

**Published:** 2015-10-07

**Authors:** Henk Pander Maat, Leo Lentz, David K. Raynor

**Affiliations:** 1 Utrecht Institute of Linguistics OTS, Utrecht University, Utrecht, the Netherlands; 2 Faculty of Medicine and Health, University of Leeds, Leeds, United Kingdom; University of Catania, ITALY

## Abstract

The structure of patient information leaflets (PILs) supplied with medicines in the European Union is largely determined by a regulatory template, requiring a fixed sequence of pre-formulated headings and sub-headings. The template has been criticized on various occasions, but it has never been tested with users. This paper proposes an alternative template, informed by templates used in the USA and Australia, and by previous user testing.The main research question is whether the revision better enables users to find relevant information. Besides, the paper proposes a methodology for testing templates. Testing document templates is complex, as they are “empty”. For both the current and the alternative template, we produced a document with bogus text and real headings (reflecting the empty template) and a real-life document with readable text (reflecting the “filled” template). The documents were tested both in Dutch and in English, with 64 British and 64 Dutch users. The test used a set of scenario questions that covers the full range of template (sub)topics; users needed to indicate the text locations where they expected each question to be answered. The revised template improved findability of information; this effect was strongest for the “filled” template with readable text. When participants were shown both filled templates, there was a clear preference for the revised template. A closer analysis of the findability data revealed question-specific effects of topic grouping, topic ordering, subtopic granularity and wording of headings. Most of these favoured the revised template, but our revision led to adverse effects as well, for instance in the new heading *Check with your doctor*. Language-specific effects showed that the wording of the headings is a delicate task. Generally, we conclude that document template designs can be analyzed in terms of the four parameters grouping, ordering, granularity and wording. Furthermore, they need to be tested on their effects on information findability, with template translations requiring separate testing. The methodology used in this study seems an appropriate one for such tests. More specifically, we find that the new patient information leaflet template proposed here provides better information findability.

## Introduction

Patient information leaflets (PILs) are an example of a text ‘genre’, i.e. a class of documents that displays a conventional structure. Most genre conventions slowly evolve, as a result of a multitude of interactions between writers and readers [1]. However, some may change abruptly as a result of regulations, and PILs are an example, as a PIL *template* has been produced by a committee of the European Medicines Agency called the ‘Quality Review of Documents’ group (the so-called ‘QRD template’) [2]. The template specifies a fixed sequence of headings, stipulates what kind of information needs to be provided under these headings, and requires particular wordings for various subtypes of information. The order of the information is stated in law, the rest is ‘guidance’ but is in practice generally enforced. Regulatory agencies assess whether PILs comply with these templates. Without approval of the PIL, the medicine cannot be admitted to the market.

In other parts of the world, similar templates apply to leaflets which accompany medicines. In the US there is a mandatory template for medicines with particular safety issues (called ‘Medication Guides’) [3]. In Australia there is a template for the Consumer Medicine Information (or ‘CMI’) leaflets supplied with all prescription and pharmacist-only medicines [4], in the form of a ‘core CMI’ for classes of drugs. Despite these templates, studies in all three continents show that patient information does not always meet peoples’ needs. A systematic review [5] found that most people did not value the written medicines information they received, they wanted sufficient detail to meet their needs, and most wanted to know about any side effect that could arise. Specific studies also show that the leaflets do not always help patients to find relevant information, to understand and use this information, to recall everything that is needed for a safe and effective use during everyday life, and that patients often do not feel motivated to read the information and to comply with the instructions [6–10].

This paper explores an alternative template for Europe. To inform this re-design, Table 1 compares the current European template with its counterparts from Australia and the US. An immediately apparent difference is that the US leaflets are mandated to have an initial heading *What is the most important information I should know about X*? under which particular risk(s) of the medicine are described. This means that this initial section lacks balance, as it focuses solely on the possible harms of the medicine. The US and Australian leaflets differ from the European leaflets in that they include a heading on matters such as eating, drinking and driving: *While you are taking* (Australia) and *What should I avoid*… (US). European leaflets present this information under specific subheadings in the *before you take* … section. Besides these differences in the *grouping* of the information in sections and in the *wording* of headings, the templates show differences in *granularity* i.e. the number of subheadings used to segment particular sections. Finally, there are differences in the *order* of the information. The US and Australian leaflets place their *while taking …* information after the usage instructions, while the European leaflet provides it before the instructions.

**Table 1:** The EU template for patient information about medicines compared with its US and Australian counterparts. US and AU templates have been reordered for comparison (but not renumbered).

EU template	US template	Australian template
	**1. What is the most important information…?**	**1. What is in this leaflet?**
**1. What X is and what it is used for**	**2. What is X?**	**2. What is X used for?**
**2. What you need to know before you <take> <use> X**		**3. Before you take X**
Do not take X if:	**3. Who should not take X?**	When you must not take it
Warnings and precautions		Before you start to take it
Children <and adolescents>		
Other medicines and X		Taking other medicines
X with food, drink and alcohol	**5. What should I avoid while taking?**	**5. While you are taking X**
Pregnancy and breast-feeding		Things you must do
Driving and using machines		Things you must not do
		Things to be careful of
This medicine contains		
**3. How to <take> <use> X**	**4. How should I take?**	**4. How to take X?**
<Use in children <and adolescents>>		How much to take
<If you <take> <use> more X than you should>		How to take it
<If you forget to <take> <use> X>		When to take it
<If you stop <taking> <using> X>		How long to take it
		If you forget to take it
		If you take too much
**4. Possible side effects**	**6. What are the possible or reasonably expected side effects?**	**6. Side effects**
<Additional side effects in children <and adolescents >		
**5. How to store X**		**7. After taking X**
		Storage
		Disposal
**6. Contents of the pack and other information**	**7. General information**	**8. Product description**
What X contains		Ingredients
What X looks like and contents of the pack		What it looks like
Marketing Authorisation Holder and Manufacturer		Manufacturer

However, the similarities between the templates are more striking. All three have an initial section identifying the medicine *What is X [used for]*? and then proceed with information on who should not take the medicine. Side effects are always placed after usage instructions. All three templates end with general information. These similarities do not appear to be based on empirical studies on optimal structures for patient information.

In a pioneering study, Morrow et al. [11] investigated the *medication scheme* of patients. They used card sorting to look at knowledge organization and text comprehension, for three fictional medicines. Participants sorted 10 sentences on cards, and these were generally sorted into two categories: “the medication and how to take it” and “potential problems associated with taking the medication and what to do if they occur.” The participants’ preferred order was “medication” (*name–purpose*), “how to take it” (*dose–schedule–duration*) and “problems” (*warnings–side effects–emergency*). In a follow-up study, three versions of instructions were presented: *compatible* (items presented in the preferred grouping and order); *category* (grouping preserved but not the order) and *scrambled* (all items in non-preferred positions). The results supported the validity of the hypothesized scheme: the scrambled version yielded significantly poorer recall scores than either of the other two. Interestingly, the preferred order differs from all three templates discussed earlier, in that it places the warnings and side effects after the usage instructions.

A subsequent study used a larger set of cards, based on the more extensive European PILs [12]. The preliminary part used a closed card sorting procedure, where participants placed cards under a set of category headings they expected to find information. The study demonstrated that the EU template structure is counter-intuitive for some scenarios, most marked for when people could drink alcohol, the time between each dose, and disposal of the medicine. Such a closed card sort study may detect mismatches between reader expectations and the template, but it does not show what an optimal PIL scheme looks like. Hence the main study was an open sorting task, where readers sorted 75 cards with sentences found in actual PILs into as many or few groups as they wanted, and assigned a name for every group they made. They then put the groups in order. The common denominators in the groups formed reflected various levels of abstraction. Cards with sentences *Do not drive when sleepy* and *Do not use machines when dizzy* were positioned in a group called *Side effects*, and breast feeding information in a group called *Pregnancy*, but also in the *Side effects* group. The side effects group also contained information about drug interactions. We concluded that in its most general interpretation, the side effects category includes various kinds of effects of the medicine in daily life and interactions with other medicines.

A second finding is that participants use an *action* frame (for example *Contact your doctor*) and a *user situation* frame (for example *Pregnancy and breast feeding*) for grouping information. A statement such as *Tell your doctor if you plan to become pregnant* was located in both groups, and hence can be framed in both ways. This seems to imply that template designers should choose one frame and stick to it. A third observation relates to some clear preferences for segments at the start and at the end of the document. Like in the Morrow et al. study, respondents prefer a first section with general information on *What the medicine is used for*, followed by *Directions for use*. A final section about *Registration data* also seems to be preferred by most respondents. In between, there is a `midfield´ that not clearly matches with the results of the Morrow et al. study. Here we find groups with information on the medicine (*Ingredients*, *packaging*, *storage*) and groups with usage information (*Do not use or take care*, *Side effects*, *Contact your doctor*, *Driving and using machines*, *Pregnancy and breast feeding)*. The usage group might be interpreted as a “problems” section in terms of Morrow et al., but the ordering of these groups could not clearly be separated from the other midfield group with a focus on the medicine. This might also be a “framing” effect: either the medicine aspect may be foregrounded or the user perspective.

The alternative template structure derived from this study is presented in Table 2, reflecting two major decisions. First, we split up the medicine information based on the results of the cluster analysis and second, we decided not to include a separate section *Contact your doctor*, because we assumed that patients will more effectively navigate a PIL that is segmented in user situations (driving, pregnancy, diabetes, allergies, etc.) than a PIL combining all these situations in one contact section. But, clearly, the proposal needed testing.

**Table 2:** Alternative template structure proposed by Pander Maat & Lentz [12].

**Medicine–goal and ingredients**
What the medicine is used for
Ingredients and medicine group
** Usage–directions**
Directions for use
** Usage–problems **
Do not use or take special care
Side effects
Driving and using machines
Pregnancy and breast feeding
**Medicine–other topics**
Packaging and appearance
Storage
Registration

Strange as it may seem, the European PIL template has never been tested as such. This is ironic as the manufacturers, who have to write the PILs based on the template, are required to user test the resulting leaflets. As our earlier work points to potential problems with the template [9;12], this study reports on the development of an alternative template, and on a comparative test of the two templates. This test focuses on the findability of information. We feel that this is the primary yardstick for evaluating text templates and more generally, for evaluating the structure of instructional text–and probably the primary functional pressure that affects the evolution of instructional text structures in real life. Hence the practical question in this paper is whether the alternative leads to better findability than the current template. We also address a general and more fundamental question: what is a reliable methodology for the empirical validation of a template for mandatory discourse in an international context?

## Materials and Methods

Testing a proposal for an improved template for PILs implies testing interventions at the four levels of text structure which have been introduced above:

- Grouping: which topics will be grouped together in one section?
- Granularity: to what extent are subtopics provided with subheadings?
- Ordering: in which sequence will the sections be presented?
- Wording: how are headings and subheadings formulated?

Our decision to split up the section on medicine information is an intervention on the level of grouping: *goal and ingredients* are separated from topics such as *packaging* and *storage*. This leads to changes on the wording level: separate headings have to be created for both sections. At the same time, a decision had to be made on the level of granularity: in the original EU template the subtopics of *packaging* and *storage* were combined under a single heading. In our proposal we decided to use separate subheadings for these subtopics, because in reader tests respondents had trouble in finding information about storage. This implies that another decision has to be made on the level of ordering: first packaging and then storage or the other way round?

The interdependence of these four dimensions has methodological consequences: a new template is always one choice out of a multitude of options. Thus, a comparative template test is not a tightly controlled experiment, as the templates will most often differ on all four levels in several sections. This means we need to look at aggregated success scores, but also need to provide a qualitative analysis focusing on explanations of differences in terms of grouping, ordering, granularity or wording choices.

On a theoretical level, we note that the effectiveness of a template derives from interactions between several constraints. A large body of experimental research exists on effects of headings on reading (e.g. [13] and [14]), but most studies confine themselves to inserting or deleting headings at particular text locations. Such studies reveal, for instance, that readers use a heading as an indicator of a change in topic, which often leads to longer reading times of the first sentence after the heading [15]; or that search times are faster in heading conditions than in no-heading conditions [16]. Generally then, higher granularity will facilitate information searching. But possibly, searching a larger number of headings will have a downside as well: more decisions will have to be taken, and new distractors are introduced. The optimal number of headings is not yet known. Moreover, no experimental work is known on grouping, ordering and wording headings. In website navigation studies it has been shown however that the overlap between reader task formulations and headings or link labels heavily determines navigation success [17].

Another issue in testing text structures is the construction of reader tasks. As usability means that the document is optimally matched to relevant reader tasks, this is a crucial issue. From a functional point of view, a user test needs a representative sample of the real-life tasks relevant for PILs. From an experimental point of view, a test needs to cover the manipulation of the template: for every change in headings, granularity, ordering and grouping a task should be created that is sensitive to this revision. We will return to the issue of task selection below.

## Materials

**Fig 1:**
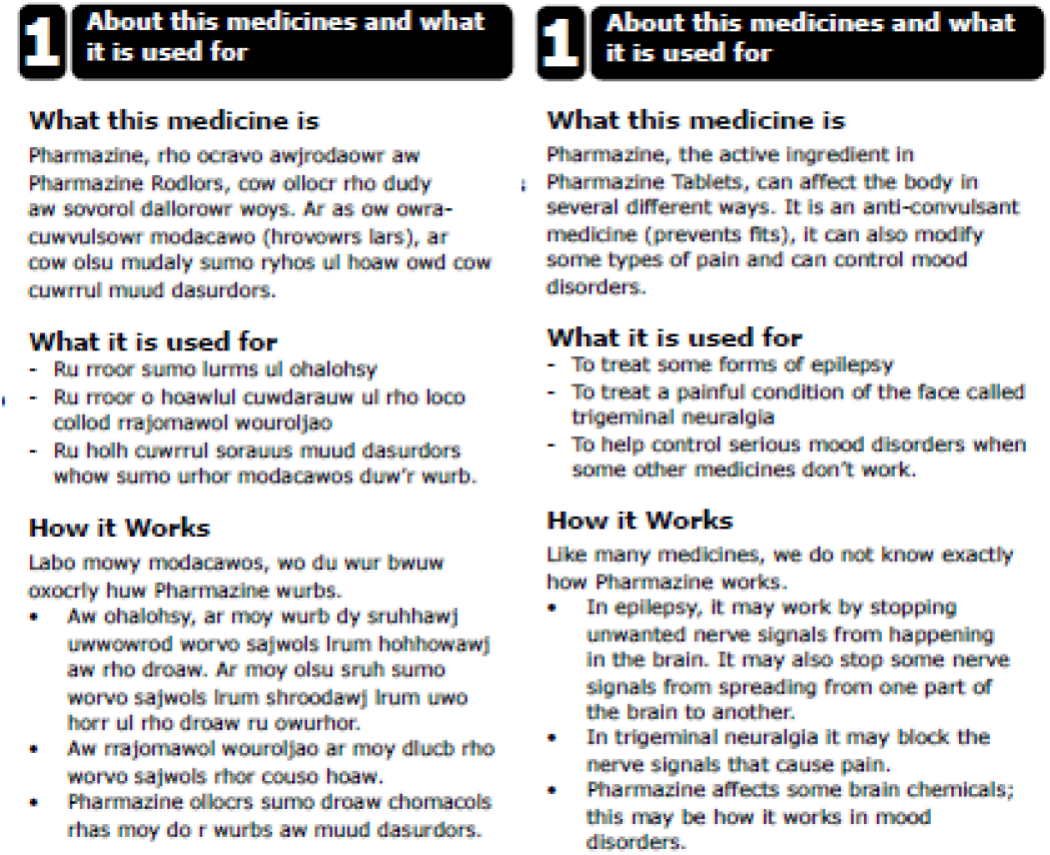
Bogus and Real text versions for the first section of the revised PIL.

Testing a template means an evaluation of the grid of a document. It can be argued that the participants should use just this grid and neglect the information that usually fills it. This is why we designed a document that looks like a normal PIL but all text–except for the headings–is presented in bogus text. Only the name of the medicine was readable. However, it can also be argued that searching should be done with a real text, as headings are meant to facilitate the finding of information in actual text. Hence we also created a PIL filled with real text. Fragments of versions are presented in Fig 1. We used an existing European PIL for Carbamazepine to create the different versions, but changed the name into Pharmazine. The real text version creates a condition with a higher ecological validity, because the experimental material looked just like a normal PIL. Thus, one variable in the design of the study was called *bogus-real*.

Starting point of the experiment was the current EU template. Thus, the revision of the template should be compared with this template. This is why we also created a *bogus* and a *real* document for the current EU PIL. The study was done in the UK and in the Netherlands, with an English and a Dutch version of these four documents. These two other independent variables were called: *version (current vs*. *revision)* and *language (Dutch vs*. *English)*. This resulted in eight different documents that were designed in Adobe InDesign CS6 with the help of professional expertise in graphic design of PILs.

Our revised template design was informed by (but not identical to) our earlier template proposal [12]:

***1. About this medicine and what it is used for***
What this medicine is
What it is used for
How it works
***2. Taking the medicine***
How to take
How much to take
When to take
How long to take
If you want to stop taking this medicine
If you forget to take
If you take too much
***3. Possible problems with this medicine***
People who cannot take this medicine
Allergies
People who should check with their doctor before taking this medicine
Possible side effects
  - Stop taking this medicine and tell your doctor straight away if you notice: (…)
  - Talk to your doctor if you have any of the side effects listed below, and they trouble you (…)
Taking X with other medicines
  - Tell your doctor if you are taking: (…)
How food, drinks and alcohol affect this medicine
Driving and using tools or machines
Pregnancy and breast feeding
Tests
***4. Packaging, storage and disposal***
Contents of the pack and appearance
Storage
Disposal
***5. Ingredients and registration***
Ingredients
Authorization holder and manufacturer

The main line of reasoning behind our revision is the following. First, the *medication scheme* proposed by Morrow et al. [11] is reflected in this revision: a first section on type of medication, then medication use and a third section on problems. But there are also sections on *packaging*, *storage and disposal* and on *ingredients and registration data*. These sections could not be integrated in that medication scheme. In the sorting results, *storage* and *registration data* clearly were located at the end of the document. The decision to connect *ingredients* with the *registration data* at the end of the document cannot be motivated by the card sorting study. In the sorting results, ingredients were strongly connected with medicine information, belonging to the first group. We decided not to follow that result in our new template, fearing that the presentation of a long list of ingredients at the start of the document would discourage patients to read the document. Indeed, a previous version of the template, which required that the ingredients were listed near the beginning of the leaflet, was widely criticized, notably by patients and patient groups, because of this [18]. As a result, this was changed in subsequent versions of the template. In the current template, as in connected genres like user instructions for technical devices, technical details are placed at the end of the document. There is a more general issue at stake here, on how to balance the needs of the minority (such as people who are allergic to, or intolerant of substances such as lactose, gluten or peanut oil) and the majority who are put off by the prominent placing of this information.

Second, the wording of headings was as informative as possible. The current EU template contains uninformative headings such as *Before you take X* and *Further information*. We ensured that every heading presented some key words of the main topic(s) in order to improve location success.

Third, we introduced higher granularity in the structure, not on the top level (current has six sections and revised has five sections) but on lower levels. On the second level the revised template has 24 sections, while the current EU template has 16 sections. We also introduced a third level, with three subsections, which is not present in the current template. As a consequence, the revised template has more specific headings (*How much to take*, *When to take*, *How long to take)*.

Fourth, we choose to frame the information as much as possible in terms of patient actions and introduced some new groups to stress that action-oriented framework (*Talk to your doctor if…*, *Tell your doctor if you are taking…*). However, some sections could not be framed in terms of actions (*Contents of the pack*, *Ingredients*).

### Questionnaires and counterbalancing

In order to test whether the revised template provides better findability than the current one, we designed a set of scenario tasks. This is a common user testing method in which participants are interviewed individually to determine whether they can find key pieces of information in a document [19–21].

We created a set of 25 tasks that covered the entire range of template topics. Every task covers a topic that is presented in the PIL, but we avoided the use of specific heading words in the task descriptions. Every task should require more than just matching words. We decided that a within-subjects design would be best to control for subject variance. Hence each subject worked with both templates. This meant that two variants had to be constructed for each task. This led to two parallel sets of 25 questions. The questions presented in both sets were designed as pairs relating to the same location in the PIL. This is illustrated in Table 3.

**Table 3:** A pair of scenario questions presented in the two different question sets relating to the same topic.

Question set	Question	Correct heading current version	Correct heading revised version
1	*Suppose you have heart problems*. *Are you allowed to use this medicine*?	Do not take Pharmazine if…	People who cannot take this medicine
2	*Suppose you have had blood problems*. *Are you allowed to use this medicine*?	Do not take Pharmazine if…	People who cannot take this medicine

In this way, scenario tasks covered the information under similar headings of the revised and current PIL but were not identical. Half of the participants answered the heart problem question in the test for the current template, the other half took this question in the test for the revised template. Thus both question sets were used for both templates. Table 4 provides the 8 cells in our counterbalanced design for both countries (each cell contains 8 participants). Duplicating the design in Table 4 of course implies creating both English and Dutch versions of the question sets. In order to avoid ordering effects, we used four orderings of the questions in each set.

**Table 4:** The eight-cell design used in both countries; participants in each cell see both a current (C) and a revised (R) template.

Condition	Combination of template version and text variant	Combination of template version with question set (QS)	Reading order template versions
1	Current–bogus	Current–QS1	1
	Revised–real	Revised–QS2	2
2	Current–bogus	Current–QS1	2
	Revised–real	Revised–QS2	1
3	Current–bogus	Current–QS2	1
	Revised–real	Revised–QS1	2
4	Current—bogus	Current–QS2	2
	Revised–real	Revised–QS1	1
5	Current–real	Current–QS1	1
	Revised–bogus	Revised–QS2	2
6	Current–real	Current–QS1	2
	Revised–bogus	Revised–QS2	1
7	Current–real	Current–QS2	1
	Revised–bogus	Revised–QS1	2
8	Current–real	Current–QS2	2
	Revised—bogus	Revised–QS1	1

In order to collect evaluations of the two versions, we used a questionnaire based on the CIRF (Consumer Information Rating Form) of Koo et al. [22] containing five items with a five point scale from ‘1’ (very easy) to ‘5’ (very difficult): ‘how easy or hard would you say the information in the leaflet is to read / understand / remember / locate information in / keep for future reference’. The readers’ evaluation concerning the layout of the PIL was measured with 15 additional items. Reliability of both parts of the questionnaire was above .80 (Cronbach’s Alpha).

The interview was concluded with a ‘split run test’ in which the interviewer presented a current and revised PIL version with real text. The participants were asked to express their preferences on a five-point scale, with the versions at both ends of the scale. That is, a strong preference for one version would be a score of either 1 or 5. The left-right positions of the two versions were counterbalanced in each participant group. Finally, the participants were asked to explain their choice.

### Participants

We recruited 128 participants: 64 UK citizens and 64 Dutch citizens. They participated voluntarily and were compensated for their participation at the end of the interview. The participants were recruited from databases of members of the public who have volunteered to take part in medicines information studies. In the UK this was through Luto Research, a university spin-out company specializing in user testing of medicine leaflets. The participants from The Netherlands came from MediLingua, a company with similar expertise. Each cell from Table 4 contained 8 participants, according to the following criteria.

- Four participants of each sex
- No more than three participants with higher education
- Two participants in each age group (20–30, 30–40, 40–50, 60–80)
- At least two participants are unemployed or retired

A general inclusion criterion was that none of the participants should be familiar with this medicine or medicines in the same medicine group. Table 5 provides demographics for the participants in both countries. There were no differences between condition either with respect to whether participants used written documents in their profession or whether they were extensive medicine users.

**Table 5:** Participant characteristics in the UK and NL samples.

	United Kingdom			The Netherlands			Total
	Male	Female	Total	Male	Female	Total	
**Sex**	32	32	64	32	32	64	128
**Age** (sd)	46 (20)	44 (17)	45 (18)	44 (18)	45 (17)	45 (17)	45 (18)
**Education**							
GSE’s (%)	6 (19)	6 (19)	12 (19)	9 (28)	11 (34)	20 (31)	32 (25)
A levels (%)	15 (47)	14 (44)	29 (45)	6 (50)	18 (56)	34 (53)	63 (49)
Grads (%)	11 (34)	12 (37)	23 (36)	7 (22)	3 (9)	10 (16)	33 (26)
**Use of written documents at work**							
Yes (%)	17 (53)	20 (63)	37 (58)	15 (47)	16 (50)	31 (48)	68 (53)
No (%)	15 (47)	12 (37)	27 (42)	17 (53)	16 (50)	33 (52)	60 (47)
**Take medicines long-term**							
Yes (%)	8 (25)	18 (56)	26 (41)	13 (40)	13 (40)	26 (40)	52 (41)
No (%)	24 (75)	14 (44)	38 (59)	19 (60)	19 (60)	38 (60)	76 (59)

This study was approved of by the School of Healthcare Research Ethics Committee, University of Leeds (reference number SHREC/RP/249), including its consent procedure, which consisted of filling out consent forms by all participants. The forms were kept on file.

### Procedure

The interviews took place in a face-to-face setting. We used a sound recording device and hard copy observation forms to score participant performance. All PIL materials and questionnaires were also in hard copy. Every test session had nine stages.

1. The procedure was explained. A scenario was presented in which the participants should imagine that they went to the doctor and were prescribed a medicine. Back home they realized that there were some questions to be answered about this medicine. In case the *bogus* leaflet was presented, the interviewer explained that the text had been made unreadable except for the headings.
2. The participants were invited to scan the leaflet for approximately one minute.
3. A structured interview was done with one of the question sets. Every question was read aloud by the interviewer and then presented on a card to avoid misunderstanding or recall problems. Participants could refer to the leaflet when locating the answer. They did not have to answer the question but just point at one of the headings. For every question, the interviewer made notes on location success and comments made by the participant or interventions of the interviewer for clarification in case of problems.
4. The participant filled out the questionnaire on evaluation of the leaflet read first.
5. The participant was invited to scan the second leaflet.
6. The participant was interviewed about this leaflet.
7. The participant evaluated the second leaflet.
8. In a split run test, a preference for one of the two leaflets was elicited.
9. The interviewer explained the aim of the test and demonstrated differences in both templates. Compensation was paid.

## Results

### Preparing the data

The wording of the tasks was examined for face and content validity; it was not pre-tested as similar questions had been used in earlier studies. However, the wording of a few tasks was found to be misleading. For instance, one of our questions runs as follows: ‘Imagine you have epilepsy. Did the doctor prescribe the right medicine for you?’ This question was meant to refer to epilepsy as the condition to be treated, but the answers revealed that many readers understood epilepsy as a possible complication interfering with the use of Pharmazine to treat another condition. We discarded three tasks which were affected by this reading in both question set variants. Another task posed a problem in one of its question set variants: ‘How many times a day should you take a dose of this medicine?’ This question was meant to lead to the heading *When to take*, but as the majority of readers chose *How much to take*, the wording promoted a dosage reading as opposed to an administration time reading. This left us with data for 21 tasks.

### Effects on locating information

Table 6 presents means and standard deviations for the total of correct locations in our 21 scenarios. Tests are two-sided unless indicated otherwise.

**Table 6:** Means and standard deviations for the total number of correct locations.

	Current (N = 128)	Revised (N = 128)
Real	15.69 (2.60)	17.27 (2.11)
Bogus	15.06 (2.50)	15.86 (2.71)

In our data set, the factors bogus-real and template version (current-revised) were coupled within subjects (a participant reading the current template with real text received the revised template with bogus text, and the other way round). As both factors affect the total of correct locations, we could not separately test them, nor their interaction, within subjects. Instead, a data set was created in which every participant contributed two observations (for each template read). Table 6 presents the means and standard deviations for locating success for both template versions and bogus-real; both are now between-subject factors.

First, we tested the effect of bogus-real for both template versions (see columns in Table 6). This analysis shows that in the revised template, the real text subjects perform better than the bogus text readers (F[1,126] = 10.71, p < .01, eta^2^ = .078). In the current version, we find no such difference.

Second, we examined template version effects (rows in Table 6) for both the bogus and real text variants. In this analysis, country and question set were used as additional variables. The analysis yields a highly significant advantage for the revised template when combined with real text (F[1,120] = 14.22, p < .001 eta^2^ = .101), as well as a modest advantage in the bogus text condition (one-sided test; F[1,120] = 2.99, p < .05, eta^2^ = .023).

There were no main effects for country and question set in either analysis. In the real text row however, we find a three-way interaction of template version, question set and country (F[1,120] = 3.97, p < .05, eta^2^ = .032), due to the fact that in the current template condition, there is an interaction between country and question set which is lacking in the revised template conditions. Probably, this interaction between language and question set reflects wording effects; we will later illustrate these.

For now we conclude that leaflet users will better find what they are looking for when using a revised template. This effect is stronger for the leaflet version in which headings are accompanied by actual text, perhaps due to a better fit between headings and text in the revision.

### A closer look at location effects

What aspects of our complex manipulation were actually responsible for the improved location performance we found? We explored this issue by analyzing our tasks questions one by one. The 128 observations for each scenario were examined in a loglinear analysis using five factors: location (correct-incorrect), template version (current-revised), question set (Q1-Q2), text variant (real-bogus) and country (Dutch-UK). In a loglinear analysis, version effects surface as location-by-version interactions. Robust version effects are found when a question only shows this two-way interaction, in the absence of higher-order interactions involving country and text variant. We will now discuss various effects that seem related to different design dimensions. Although the revision was globally successful, some differences favoured the old template and others the revised version.

A *grouping* effect was shown in a question about the wish to stop using the medicine (19). In the revised template there is a large section titled *Problems with this medicine*, including subsections such as *Check with your doctor before taking the medicine* and *Possible side effects*, which contained the lower order section *Talk to your doctor if you have any of the side effects listed below*. Readers of the revised template tended to go to these sections when confronted with a question about the wish to stop taking the medicine, probably because they thought the patient in this scenario was having ‘problems with the medicine’. The correct heading for this scenario is the section *Taking the medicine*, which includes the subsection *If you want to stop taking this medicine*. But the association between stopping and taking the medicine is clearly not a natural one. The two newly marked ‘problem’ subsections proved powerful distractors for this scenario, resulting in better location for the current template (Z = -2.64 p < .01 SE = .092).

An *ordering* effect of our intervention was found in a task that introduced scenarios involving pregnancy and breast feeding. The heading *Pregnancy and breast-feeding* has not been revised in the alternative template. However, in the current template this subheading is placed in the second section of the main heading *Before you take this medicine*, while in the revised template it is at the end of the long new section *Possible problems with this medicine*. This led a number of readers to incorrectly locate the information under our new heading *People who should check with their doctor before taking this medicine*, which is at the beginning of this section. The current template had higher location scores for this question (Z = -2.52 p < .05 SE = .074). Information that is presented further away from a main heading is located less well.

For five tasks, the revised template seems superior due to increased *granularity*. For instance, a question about ‘check-ups’ or ‘examinations’ during treatment is directly accommodated for by the added subheading *Tests* in the revised template (Z = 5.69 p< .001 SE = .082). Another question is directly associated with the added subheading *How long to take* (Z = 5.23 p < .001 SE = .119). And a question about what to do with unused medications, is catered for by the added *Disposal* subheading (Z = 2.87 p < .01 SE = .136).

Increased granularity may have its downside however. Added headings gave problems for a task that asked about ‘the time during the day at which a dose of the medication should be taken’. In the current template, this issue is not specifically addressed in a subheading, so that nearly all readers of the current template located it under the main heading *How to take Pharmazine*. In the new template, four subheadings (that can also be found in the Australian template) have been added to this section: *how to take*, *how much to take*, *when to take* (correct) and *how long to take*. Higher granularity forces participants to choose between these subheadings. About 15% of the participants makes the wrong choice, which leads to a better score for the current template (Z = -2.24 p < .01 SE = .184). Some readers locate the required information under *how much to take*. With the benefit of hindsight, we can see why. First of all, the question mentions dosage and not only timing. Second, in actual PILs, instructions often combine information on dosage and timing, such as in ‘take two tablets with every meal’. Hence a future template should probably combine the headings, as in *how much and when to take*.

Ten tasks showed *wording* effects. For instance, the first scenario asked about the composition of the medicine; it uses the English word *component* and the Dutch word *bestanddeel*. The Dutch term is only used to refer to chemical substances, whereas *component* has a more general meaning. This may explain why the Dutch readers more often located this information under the heading *Medicine ingredients* than the English readers (Z = 2.33 p < .05 SE = .064). This is the wrong choice however, as the task relevant information is not in that section but in section 1 about what the medicine is. Similarly, another task had a question that referred to allergies for certain types of food components, something that also needed to be checked in the revised *Allergies* heading or the current heading *People who cannot use this medicine*. Because of the reference to food however, many readers incorrectly located the food allergy question under the heading about combining food and drinks with the medicine. In both cases, the wording of a scenario directly affected location success.

To sum up, a closer look at the data revealed specific effects of grouping, ordering, granularity and wording. Sometimes these effects were in favor of the revised template, but there were adverse effects as well. The creation of the *check with your doctor* and *talk to your doctor* groups produced mainly adverse effects for the revised template, attracting readers that would like to talk with their doctor on quite a number of the questions we offered. Changes in the order of segments had direct effects on localization as “early” segments attract more readers than “late” segments. Increased granularity had largely positive effects: more specific headings and subheadings may lead to a closer match between scenario and text fragment; in some cases, more signposts present readers with more difficult choices. Finally, the wording of headings is a delicate task for the template designer. Several differences between Dutch and English participants could be explained in terms of the wording of headings. This suggests that translations of template headings always need to be tested carefully.

### Version preferences

In the split-run test, participants were shown both templates in the Real version. They were invited to make a choice on a scale from one (current) to five (revised). There were no differences between Dutch and English participants in these scores. A clear preference was found for the revised template: 68% vs 32% (N = 119), when we look at scores on the extremes of the scale (5 and 1). A further analysis of the scores on different attitude scales did not show any differences between versions.

The preferences were motivated in an open field. We coded all motivations as belonging to one of four categories: grouping, order, granularity and wording of headings. Comments on other aspects and unclear comments constituted a fifth category. The coding was done separately by both first authors, resulting in a Cohen’s Kappa of .67. Differences were resolved in a conservative direction, meaning that most controversial comments ended up in the ‘unclear’ category. A chi-square test showed a significant difference in the nature of the motivations (Chi^2^ = 11.36, df = 3, p < .05) that was primarily due to the fact that 21% of the motivations in favour or the revised template mentioned the (larger) number of headings, whereas those preferring the current version never mentioned the number of headings. An illustrative quote from the comments is: *it is clearer because of the sub-headings*; another participant was positive about the *smaller chunks of information* in the revised version.

## Discussion

Based on earlier work published by Morrow et al. [11] on medication schemes, we set out to test the current EU template for patient information leaflets. In earlier work [9;12] we identified problems patients may experience with this template and we found indications for alternative structures. We designed an alternative PIL template and tested both the current and the revised template in the United Kingdom and the Netherlands. Participants (N = 128) were asked to locate information using scenarios presented in different question sets. The practical conclusion of this paper is that, although not all scenario questions showed improvements, the revised template enables readers to locate information more effectively.

A question specific analysis revealed effects of manipulations on different levels. Introducing new groups may lead to a loss in performance on existing sections of the document. Changing the order of segments will effect localization of segments that are presented further away from the main heading. Higher granularity often leads to gains (better matches between scenarios and headings) but shows some losses as well. And wording changes in headings may have unexpected adverse effects. Thus, the design and the revision of any template is a delicate operation.

Testing the design of templates is complex as well, because readers looking at patient information leaflets do not see templates but complete documents. Hence we created two versions, a real PIL with information about a specific medicine and a ‘bogus’ document with readable headings and unreadable text. Thus, we tried to avoid the criticism that gains of one template could be caused by the wording of text fragments. Indeed, the data demonstrated a “text effect”: in the revised template location success was higher for readers who could read the complete document than for readers who could only read the headings. For the current template no such effect was found. This means that the interpretation of the headings was more successful when respondents were able to connect their understanding of the revised headings with the text fragments connected with these headings. Of course, ecological validity is higher in the “real text” condition, because the test document is a realistic example of everyday patient information. However, the superiority of the alternative template was also found in the “bogus” condition, which means that the effect is robust. For further template tests, we would advise to use both real text and blurred or bogus presentation formats.

Another methodological issue was the introduction of two question sets with similar scenario questions in both the Dutch and the English settings and for the different conditions. We did find an interaction of question set with country for the current template. For one of the question sets Dutch participants—reading the current template—performed better than English participants. This might be interpreted as a translation issue. Within language areas, there were no interactions involving question sets; this increases external validity for our findings.

When we compare the different templates used in Europe, the US and Australia, some remarks can be made on strengths and weaknesses. All templates start by identifying the medicine and end with manufacturer information. Both decisions are supported by our card sort data and by the results of Morrow et al. [11].

The templates are also similar in where they provide the side effects: after the instructional *How to take* section. Our findability study is inconclusive here, as it does not demonstrate clear ordering effects for the side effects information.

The headings for the instructional sections are well chosen: *How to take X*? (EU and AU) and *How should I take*? (US). Given that some medicines have extremely long or difficult names (e.g. Bendroflumethiazide), we prefer the US heading without the name of the medicine in the title of the section.

Every template has some weaknesses. One weakness is long headings. The Australian template has the shortest heading for the side effects section: *Side effects*. The US template heading is: *What are the possible side effects*? In their navigation process through the document patients do not need such explicit variants, as long as the topic question at stake is clear. The EU template has a second heading *What you need to know before you take X*, which again has possible long medicine names in the long title. The Australian template heading for a comparable section is: *Before you take X*.

Another weakness is what we call “empty headings”. The US template has a final heading *General information about the safe and effective use of X* and the EU template has: *Contents of the pack and other information*. Patients will not be able to construct any expectation on the contents of general or other information. In our reader tests, such empty headings serve as “false friends” for patients having trouble in finding information. They may hope to find it in this section, but are often disappointed.

The Australian and US templates present a generally headed separate *while taking* or *what should I avoid* section, which presents information on eating, drinking, using machines and pregnancy. We did not directly test this in our findability study, but the subheadings for this information in the European leaflets present no findability problems.

More generally, the Australian PIL structure is chronologically oriented: the order is *before using–while using–after using*. This temporal structure seems not necessary for better findability, and at times it does not really fit the information. For instance, storage of the medicine is relevant during use, while disposal is relevant after use; yet the Australian template presents both topics under *After taking X*. Likewise, the side effects seem to be relevant while using the medicine, but are in a separate section.

Our findability study presents clear gains for increased numbers of subheadings for five tasks. By and large, the greater granularity in the Australian template is supported in our study, but some of the new subdivisions need to be tested as they may backfire, which was illustrated for the potentially confusing distinction between ‘when’ and ‘how much’ to take.

Text structures for patient information should map with patients’ cognitive ways of grouping and labelling information, and thus with their expectations about what information appears where in the text. This is important, because it is not so much *understanding* as well as *finding* information that should be facilitated by a mandated structure. Our findability study has shown improvements for the revised template, but has also revealed a number of imperfections that need to be addressed in a newer version of it. We have demonstrated the beneficial and adverse effects of grouping decisions, ordering of information, granularity of the structure and the wording of headings. These findings will be used to further improve the new structure. Elsewhere [23], we propose a new template that is maximally compatible with the evidence amassed so far. Such proposals for internationally mandated text structures, we suggest, should be tested using the methodology that has been pioneered in this study: using different languages and balancing bogus and real text versions.
